# Total Hip Arthroplasty: The Influence of Different Management Structures in Germany and the Netherlands on Preoperative Expectations, Self-Efficacy, and Postoperative Satisfaction

**DOI:** 10.7759/cureus.84088

**Published:** 2025-05-14

**Authors:** Yvet Mooiweer, Roy Stewart, Baukje Dijkstra, Robert Oosterveld, Martin Stevens, Gesine H Seeber

**Affiliations:** 1 Department of Health Services Research, School of Medicine and Health Sciences, Carl von Ossietzky Universität Oldenburg, Oldenburg, DEU; 2 Department of Orthopedics, University Medical Center Groningen, University of Groningen, Groningen, NLD; 3 Department of Health Sciences, University Medical Center Groningen, University of Groningen, Groningen, NLD; 4 Department of Orthopedics, Frisius Medical Center, Leeuwarden, NLD; 5 Department of Radiology, Deventer Hospital, Deventer, NLD; 6 Division of Orthopedics at Campus Pius-Hospital Oldenburg, School of Medicine and Health Sciences, Carl von Ossietzky Universität Oldenburg, Oldenburg, DEU

**Keywords:** healthcare structures, hip prosthesis, osteoarthritis, postoperative outcome, preoperative expectations

## Abstract

Objective

Total hip arthroplasty (THA) is considered a highly effective treatment for end-stage hip osteoarthritis (OA). THA management in Germany and the Netherlands significantly differs. This study aims to give a first glimpse of the extent to which the way of management in both countries influences patients’ preoperative expectations, self-efficacy, and, consequently, their postoperative satisfaction.

Methods

This was a transnational, prospective, multicenter observational study that included a cohort of primary THA patients from Germany and the Netherlands. Preoperatively, patients completed the Hospital for Special Surgery Hip Replacement Expectations Survey (HSS-HRES), Self-Efficacy of Rehabilitation questionnaire (SER), and the Hip disability and Osteoarthritis Outcome Score (HOOS). Postoperatively, patients rated their satisfaction on a Likert scale (0-10). Scores of German and Dutch patients were compared using student t-tests and Chi-square tests. Simple and multiple regression analyses were used to assess the relationship between preoperative expectations and self-efficacy on postoperative satisfaction with country as the categorical variable.

Results

A total of 138 German and 152 Dutch participants were included. German patients showed significantly higher levels of preoperative expectations and self-efficacy. In the simple regression analysis, preoperative self-efficacy showed a statistically significant relationship with postoperative satisfaction (SER: B 0.129 (95%CI 0.004 - 0.255), p=.044). In the multiple regression analysis, preoperative HOOS Sport and HOOS QOL (Quality Of Life) showed a statistically significant relationship with postoperative satisfaction (HOOS Sport: B -0.020 (95%CI -0.040 to -.001), p=.042; HOOS QOL: B 0.025 (95%CI 0.002-0.048), p=.034). The country had no influence.

Conclusion

There is a difference in preoperative expectations and self-efficacy between German and Dutch patients. Moreover, preoperative self-efficacy influences postoperative satisfaction. However, this effect disappeared in the multiple regression analysis, nor was the country of treatment influential. On the other hand, we do see that higher preoperative scores on HOOS Sports and HOOS QOL lead to higher postoperative satisfaction.

## Introduction

Total hip arthroplasty (THA) is a highly effective treatment for end-stage hip osteoarthritis (OA). However, no international standard for THA management [1,2] exists, leading to varying treatment structures across countries. In that context, Germany and the Netherlands provide interesting examples as neighboring countries. Concerning THA, Germany and the Netherlands rank among the top OECD countries, with rates of 315 and 250 hip replacement operations per 100,000 population in 2019 [3]. In 2023, 273,737.00 THAs were performed in Germany [4] and 42,906 THAs in the Netherlands [5]. In the coming decades, these numbers are expected to rise substantially due to the rising numbers of OA [6].

In relation to THA management, it can be observed that the approach to care in Germany and the Netherlands differs in its organization [7]. After a two-to-three-day hospital stay, Dutch patients are generally sent home with a recommendation for physiotherapy [8]. However, as physiotherapy is not reimbursed by Dutch basic health insurance, most patients have additional insurance for a specified number of sessions [9]. By contrast, German patients can attend a 3-week rehabilitation program at a specialized rehabilitation center after a week-long hospital stay, and additional physiotherapy can be prescribed when considered indicated following discharge from the rehabilitation center. All costs are covered by the patient’s health insurance [10,11].

In THA, postoperative patient satisfaction is one of the most relevant outcomes. Both preoperative expectations and self-efficacy are associated with postoperative satisfaction and are considered relevant factors in the postoperative rehabilitation process [12-14]. Based on previous research, it is known that how a treatment is organized can influence patients’ preoperative expectations [13]. Patients with positive expectations are more likely to engage in positive health and illness-coping behavior [15,16], thereby triggering a self-fulﬁlling prophecy [17]. Self-efficacy was defined by Bandura as ‘the conviction that one can successfully execute the behavior required to produce the outcome’ [18]. Higher levels of preoperative self-efficacy predict better recovery from THA [13].

The question, thus, arises as to whether variations in THA management prompt differences in preoperative expectations and self-efficacy and, eventually, postoperative satisfaction. An answer to this question would contribute to improving the management of THA. To date, only one study by Lingard et al. [19] has compared the association between preoperative expectations and outcomes across various countries, including patients from the United States, the United Kingdom, and Australia. However, these authors focused on patients who had undergone total knee arthroplasty (TKA). This study found that Australian patients had higher preoperative expectations of better functioning after surgery; however, no differences were found regarding postoperative outcomes between the countries.

The current study aims to examine the influence of the THA management structures in Germany and the Netherlands on patients’ preoperative expectations, self-efficacy, and, eventually, postoperative satisfaction.

## Materials and methods

A transnational prospective multicenter observational study in a cohort of primary THA patients was conducted at the University Hospital for Orthopedics Pius-Hospital, Medical Campus University of Oldenburg, Germany (further referred to as Pius-Hospital) and Frisius Medical Center (FMC) in the Netherlands. The study was approved by the local METc boards in Germany (Medical Ethical Committee of the Carl von Ossietzky Universität Oldenburg, Germany (# 2018/078 and 2023-082) and the Netherlands (Medical Ethics Review Board of University Medical Center Groningen (#2018/293)).

Procedure

All patients included in the study had an indication for primary THA due to hip OA and were consecutively enrolled if they were 18 years or older. Exclusion criteria involved undergoing revision surgery, previous THA surgery on the contralateral hip, any lower limb surgery less than a year ago, or not being proficient in either German or Dutch.

Eligible patients were recruited from Pius-Hospital and FMC from 1-1-2019 till 1-1-2023. In Germany, eligible patients were informed about the study during consultation with their orthopedic surgeon and were asked to consent to participate. Patients who agreed signed an approved written informed consent form. Following consent, a questionnaire was distributed to the patients, and they were asked to complete it and return it to the research assistant either in person or by postal mail. In the Netherlands, eligible patients were sent a questionnaire by postal mail after being placed on the waiting list for surgery. Returning the completed questionnaire was regarded as informed consent. German patients received all questionnaires in German, while Dutch patients received them in Dutch.

Questionnaire

The questionnaire comprised the following: (1) inquiries related to the socio-demographic status, including age, sex, living situation (alone; together), and level of education (low; middle; high); (2) the Hospital for Special Surgery Total Hip Replacement Expectations Survey (HSS-HRES); (3) the Self-efficacy of Rehabilitation questionnaire (SER); (4) the Hip disability and Osteoarthritis Outcome Score (HOOS); and (5) a single-item question measuring postoperative satisfaction on a Likert scale ranging from 0 (i.e., completely dissatisfied) to 10 (i.e., completely satisfied) as is common practice [20]. 

The HSS-HRES was developed to determine patient expectations before surgery [21]. It comprises 18 items related to symptoms, physical activity, work, and psychological well-being. For each item, the following response format is used: ‘complete improvement or back to normal’, ‘a lot of improvement’, ‘a moderate amount of improvement’, ‘a little improvement’, or ‘this expectation does not apply to me/I do not have this expectation’. The total score ranges from 0 to 72 and is then converted into a 100-point scale where a higher score indicates higher expectations. The German and Dutch language versions exhibit good test-retest reliability and content validity [22].

The 12-item SER was developed by Waldrop and Lightsey [23]. This measure assesses patients’ beliefs regarding their ability to perform activities typically required during rehabilitation after hip or knee surgery. The SER is structured hierarchically based on item complexity. In addition, items measuring behavior in varying therapy situations, such as when pain or emotional stress occurs during rehabilitation, are included. Scoring is done on an 11-point Likert scale ranging from 0 (i.e., I cannot do it) to 10 (i.e., certain I can do it). The total score is determined by summing up the scores on the items and subsequently dividing by the number of items, resulting in a scoring range from 0-10 [24]. The original English SER has demonstrated good psychometric properties [23]. Similarly, the Dutch and German SER versions have shown high internal consistency with Cronbach’s α values of 0.94 and 0.95, respectively. The German and Dutch SER have also demonstrated good construct- and criterion-related validity [14,25]. 

The HOOS was used as a disease-specific Patient-Reported Outcome Measure (PROM) [26] to get an impression of self-perceived physical functioning. It includes five subscales: pain (10 items), other symptoms (five items), activities of daily living (17 items), sport and recreation (four items), and hip-related quality of life (four items). Standardized response options are given where each question is scored from 0-4 (i.e., a 5-point Likert scale). Consequently, a normalized score, ranging from 0-100, is calculated for each subscale (0 = extreme symptoms; 100 = no symptoms). The HOOS subscale scores are calculated by dividing the mean of the observed items within each subscale (e.g., symptoms) by four and then multiplying by 100. This number is then subtracted from 100, indicating that a lower score implies a worse condition for the patient. The HOOS format is easy to use and takes 10 minutes to complete. The Dutch and German versions are valid and reliable [27,28].

Statistical analysis

Descriptive statistics reporting the mean, standard deviation, frequency, and percentages were used to describe sample baseline characteristics and the PROMS outcomes for the German and Dutch patients. Student t-tests and Chi-square tests were employed to test for differences.

In the dataset, two groups of patients could be distinguished, one from Germany and the other from the Netherlands. Both groups were asked a question on postoperative satisfaction with a range of 0 (dissatisfied) to 10 (very satisfied) as a planned numeric rating scale (NRS). Accidentally, in the German dataset, the first 36 patients were asked whether they were satisfied or not using a yes (1)/no (0) question instead of the planned 0-10-NRS. Eventually, in the context of this study, these yes/no responses were transformed to the range of 0 to 10 by the process of multiple imputation. The following assumption was made: 0 covers the range of 0 to 5 on the planned NRS, and 1 covers the range of 6 to 10. Therefore, patients who scored 0 were added to the donor pool of patients in Germany who scored between 0 and 5. The remaining patients who scored 1 were then added to another donor pool of those patients with values ranging from 6 to 10 on the NRS. A third donor pool was derived from Dutch patients, with missing values from this subgroup able to be imputed. The implementation of multiple imputations was conducted separately by group; this was the framework employed for creating the multiple imputations for the missing data [29]. We employed the multiple imputation method under the assumption of missing at random to generate 25 multiply imputed datasets, subsequently analyzing them to ascertain potential discrepancies. To analyze the relation between preoperative expectations, self-efficacy, and postoperative satisfaction, linear regression analyses were used. A dummy variable for the simple and multiple linear regression models was created for the education variable (low, middle, and high as reference). No collinearity was found within the dataset; thus, all variables were included in the models using the enter method.

Statistical analyses were performed using SPSS version 29 (IBM Corp., Armonk, USA) and SAS version 9.2 (SAS Institute Inc., Cary, USA). The significance level was set at α=.05.

## Results

Demographic characteristics

138 German and 152 Dutch participants were included. Table 1 shows the demographic characteristics of the population. Age, sex, preoperative functional status, and postoperative satisfaction were not statistically significantly different between the two countries. However, preoperative self-efficacy and expectations were, with German patients showing significantly higher levels of both factors (Table 1). 

**Table 1:** Demographic characteristics SD=Standard Deviation; n=number participants; Χ²=Chi-square test, HOOS=Hip disability and osteoarthritis outcome Scale; ADL=Activities of daily living; QOL=Quality of life, SER=Self-Efficacy in Rehabilitation outcome scale, HSS-HRES=Hospital for Special Surgery Total Hip Replacement Expectations Survey

Variable	Total mean (SD)	German mean (SD)	Dutch mean (SD)	p-value, T-test/Χ²
Age n=290/138/152	68.9 ± 9.8	68.4 ± 10.4	69.5 ± 9.3	.356
Sex (female) (%) n=290/138/152	62.8	59.4	65.8	.263
Living situation (alone/together) (%) n=290/138/152	27.6/72.4	31.9/68.1	23.7/76.3	.119
Education level (low/middle/high) (%) n=289/138/151	34.6/39.1/26.3	32.6/34.8/32.6	36.4/43.0/20.5	.062
HOOS Symptoms (0-100) n=290/138/152	37.5 ± 17.0	36.7 ± 15.7	38.3 ± 18.3	.415
HOOS Pain (0-100) n=290/138/152	37.1 ± 14.8	36.6 ± 13.6	35.5 ± 15.8	.580
HOOS ADL (0-100) n=289/138/152	35.9 ± 15.0	36.2 ± 14.2	35.6 ± 15.8	.753
HOOS Sport (0-100) n=288/138/150	18.8 ± 17.1	18.3 ± 14.3	19.2 ± 19.4	.668
HOOS QOL (0-100) n=290/138/152	21.2 ± 13.9	21.1 ± 13.2	21.2 ± 14.5	.952
SER (0-10) n=289/138/151	7.06 ± 1.76	7.36 ± 1.83	6.89 ± 1.76	.006
HSS_HRES (0-100) n=288/138/150	73.4 ± 19.3	82.8 ± 12.5	64.6 ± 20.4	< .001
Satisfaction (0-10) n=263/94/142	8.31 ± 1.76	8.41 ± 2.07	8.23 ± 1.52	.437

Relationship between preoperative factors and satisfaction

The results of the simple regression analysis based on imputed data are shown in Table 2. Only preoperative self-efficacy showed a statistically significant relationship with postoperative satisfaction (SER: B 0.129 (95%CI 0.004 - 0.255), p=.044). The results of the simple regression analysis based on the original data are available in the Appendices. In this analysis, preoperative self-efficacy also showed a statistically significant relationship with postoperative satisfaction (SER: B 0.163 (95%CI 0.032 - 0.294), p=.015).

**Table 2:** Results of the simple regression analysis with satisfaction as dependent variable (based on imputed data) HOOS=Hip disability and osteoarthritis outcome Scale; ADL=Activities of daily living; QOL=Quality of life; SER=Self-Efficacy in Rehabilitation outcome scale; HSS-HRES=Hospital for Special Surgery Total Hip Replacement Expectations Survey

Parameter	Estimate	95% Confidence intervals		t	p-value
Age	-0.010	-0.034	0.014	-0.81	.420
Sex, reference female	0.418	-0.031	0.866	1.83	.068
Living situation, reference alone	0.060	-0.441	0.560	0.23	.816
Education level (low), reference high	0.033	-0.579	0.645	0.11	.916
Education level (middle), reference high	-0.098	-0.673	0.477	-0.34	.738
HOOS Symptoms		-0.013	0.013	-0.03	.977
HOOS Pain	0.006	-0.009	0.021	0.74	.461
HOOS ADL	0.005	-0.009	0.020	0.72	.470
HOOS Sport	-0.003	-0.016	0.009	-0.53	.597
HOOS QOL	0.012	-0.004	0.028	1.48	.140
SER	0.129	0.004	0.255	2.02	.044
HSS-HRES	0.006	-0.006	0.017	0.94	.345
Country, reference Germany	-0.138	-0.605	0.330	-0.58	.562

Table 3 shows the results of the multiple regression analysis based on imputed data. Preoperative HOOS Sport and HOOS QOL showed a significant relationship with postoperative satisfaction, though the effect was small (HOOS Sport: B -0.020 (95%CI -0.040 to -0.001), p=.042; HOOS QOL: B 0.025 (95%CI 0.002-0.048), p=.034).

**Table 3:** Results of the multiple linear regression analysis with satisfaction as dependent variable (based on imputed data) HOOS=Hip Disability and Osteoarthritis Outcome Scale; ADL=Activities of Daily Living; QOL=Quality of Life; SER=Self-Efficacy in Rehabilitation Outcome Scale; HSS-HRES=Hospital for Special Surgery Total Hip Replacement Expectations Survey

Parameter	Estimate	95% Confidence intervals		t	p-value
Intercept	7.367	4.741	9.993	5.50	< .0001
Age	-0.004	-0.030	0.022	-0.30	.761
Sex, reference female	0.417	-0.054	0.889	1.73	.082
Living situation, reference alone	-0.126	-0.647	0.394	-0.48	.635
Education level (low), reference high	0.190	-0.435	0.815	0.60	.551
Education level (middle), reference high	-0.051	-0.628	0.525	-0.17	.862
HOOS Symptoms	-0.006	-0.024	0.011	-0.72	.474
HOOS Pain	0.005	-0.025	0.035	0.33	.739
HOOS ADL	0.003	-0.028	0.034	0.20	.839
HOOS Sport	-0.020	-0.040	-0.001	-2.03	.042
HOOS QOL	0.025	0.002	0.048	2.11	.034
SER	0.103	-0.030	0.236	1.52	.129
HSS-HRES	0.002	-0.011	0.016	0.33	.742
Country, reference Germany	0.021	-0.498	0.540	0.08	.937

## Discussion

This study’s results suggest a difference in preoperative expectations and self-efficacy between German and Dutch THA patients. Moreover, from the results of the simple linear regression, it can be concluded that preoperative self-efficacy influences postoperative satisfaction. However, this effect disappeared in the multiple regression analysis, and neither country of treatment was of influence. This is contrary to the fact that German patients receive a much more extensive treatment than Dutch patients [10]. On the other hand, we do see that overall higher preoperative scores on HOOS Sports and HOOS QOL lead to higher postoperative satisfaction. 

Looking at the differences between both countries, it appears that German patients have significantly higher preoperative expectations compared to Dutch patients. A possible explanation may be that, due to the more liberal system of surgeon/hospital selection in Germany, German patients often seek out the highest-rated surgeons and hospitals for their surgical procedures. In contrast, in the Netherlands, opting for the nearest available hospital is more or less a common practice. It stands to reason that individuals who seek care from what they perceive to be the most proficient surgeon/hospital will have elevated expectations regarding the postoperative result. Another possible explanation could be that due to the German management around THA, in which patients receive very intensive and close therapeutic care from the beginning of the treatment until the end of the specialized, structured rehabilitation, expectations of the final outcome are correspondingly high [30]. This is contrary to Dutch patients who have to take more responsibility independently [10]. The arguments above can also be applied to explain why German patients scored higher on self-efficacy. The well-organized structure of THA management in Germany may foster a sense of assurance in patients regarding their surgical and rehabilitative outcomes. This may substantially heighten their sense of self-efficacy prior to surgery compared to Dutch patients.

The results of the simple linear regression reveal that preoperative self-efficacy influences postoperative satisfaction. However, this effect disappeared in the multiple analysis. This analysis demonstrated that preoperative scores on HOOS sports and QOL subscales were positively related to postoperative satisfaction. Furthermore, the absence of multicollinearity was confirmed. This outcome aligns with earlier research, showing that preoperative physical functioning and mental health positively relate to functional outcomes after THA [31]. In the systematic review of Mooiweer et al. [12], it was reported that associations between self-efficacy and postoperative satisfaction were not yet sufficiently investigated; however, in the same review, the authors also mentioned that several studies' results suggest a positive association between self-efficacy and overall outcome. Nevertheless, this systematic review included not only THA but also TKA.

To the best of our knowledge, this is one of the few studies investigating whether variations in treatment organization prompt differences in preoperative expectations and self-efficacy and, eventually, postoperative patient satisfaction. This study will be followed by a larger prospective study, entitled “Hip Across,” the objective of which is to investigate more deeply the influence of health system and patient characteristics on expectations and outcomes in THA patients in the Northern Dutch-German border region [30]. That study will present a mixed-method design, incorporating a quantitative longitudinal paired design, where patients will complete questionnaires at three distinct time points: preoperatively and 3 and 6 months postoperatively.

The current study’s results align with the study of Lingard et al. [19]. However, their study was performed on TKA patients. Their findings suggest that Australian patients had higher expectations of better functioning after surgery compared to patients from the United States and the United Kingdom. However, no differences were found regarding postoperative outcomes between the countries. In our study, we saw a similar pattern in which preoperative differences in expectations and self-efficacy between Germany and the Netherlands did not result in differences in postoperative satisfaction. Similarly, Füssenich et al. [10] found no differences in postoperative satisfaction 12 months after THA between German and Dutch patients. An additional strong point of our study was the use of psychometrically well-evaluated assessment tools in both the German and Dutch languages [17,32]. A major constraint of the present study was the presence of missing data. Yet, to address this limitation, a comprehensive process of data imputation was employed with the help of an experienced statistician (RS). This approach was necessitated by the observation that, in the German dataset, the initial 36 patients were inadvertently presented with a dichotomous question (i.e., a "yes/no" response) to assess their satisfaction. This method was employed instead of the intended 0-10 NRS. Consequently, the results obtained through this process were imputed, with specific constraints implemented to mitigate the impact on the study's outcomes. 

## Conclusions

In conclusion, based on the current study's results, the different ways in which THA management is organized in Germany versus the Netherlands seem to influence preoperative expectations and self-efficacy. Yet eventually that does not lead to differences in postoperative satisfaction. Overall, preoperative quality of life and functioning in sports do have a positive association with postoperative satisfaction.

## Appendices

**Table 4:** Results of the simple regression analysis with satisfaction as dependent variable (based on non-imputed data) HOOS = Hip Disability and Osteoarthritis Outcome Scale; ADL = Activities of Daily Living; QoL = Quality of Life; SER = Self-Efficacy in Rehabilitation Outcome Scale; HSS-HRES = Hospital for Special Surgery Total Hip Replacement Expectations Survey

Parameter	Estimate	95% Confidence intervals		t	p-value
Age	-0.010	-0.034	0.013	-0.873	.383
Sex, reference female	0.453	-0.013	0.919	1.914	.057
Living situation, reference alone	-0.129	-0.628	0.370	-0.510	.370
Education level (low), reference high	-0.154	-0.754	0.447	-0.504	.615
Education level (middle), reference high	-0.331	-0.922	.259	-1.105	.270
HOOS Symptoms	0.003	-0.011	0.016	0.399	.690
HOOS Pain	0.006	-0.009	0.021	0.751	.453
HOOS ADL	0.006	-0.008	0.021	0.837	.403
HOOS Sport	-0.003	-0.016	0.010	-0.433	.665
HOOS QOL	0.013	-0.003	0.028	1.607	.109
SER	0.163	0.032	0.294	2.458	.015
HSS-HRES	0.006	-0.006	0.017	0.991	.323
Country, reference Germany	-0.182	-0.644	0.279	-0.779	.437

## References

[1] Coulter CL, Scarvell JM, Neeman TM, Smith PN (2013). Physiotherapist-directed rehabilitation exercises in the outpatient or home setting improve strength, gait speed and cadence after elective total hip replacement: a systematic review. J Physiother.

[2] Lowe CJ, Davies L, Sackley CM, Barker KL (2015). Effectiveness of land-based physiotherapy exercise following hospital discharge following hip arthroplasty for osteoarthritis: an updated systematic review. Physiotherapy.

[3] Organisation for Economic Co-operation and Development (OECD) (2021). Health at a Glance 2021: OECD Indicators. OECD Publications, Paris; 2021.

[4] Statistischer Bericht (2025). Statistischer Bericht - Fallpauschalenbezogene Krankenhausstatistik (DRG-Statistik) - Operationen und Prozeduren der vollstationären Patientinnen und Patienten in Krankenhäusern (4-Steller) - 2023 [Statistical Report - Case-based Hospital Statistics (DRG Statistics) - Operations and Procedures of Inpatients in Hospitals (4-digit Code) - 2023]. https://www.destatis.de/DE/Themen/Gesellschaft-Umwelt/Gesundheit/Krankenhaeuser/_inhalt.html#_69yfwu1ce..

[5] LROI: 92.491 registraties in 2023 (2025). LROI Report 2024. LROI.

[6] Rupp M, Lau E, Kurtz SM, Alt V (2020). Projections of primary TKA and THA in Germany from 2016 through 2040. Clin Orthop Relat Res.

[7] Wijnen A, Seeber GH, Dietz G (2023). Effectiveness of rehabilitation for working-age patients after a total hip arthroplasty: a comparison of usual care between the Netherlands and Germany. BMC Musculoskelet Disord.

[8] Oosting E, Kapitein PJ, de Vries SV, Breedveld E (2021). Predicting short stay total hip arthroplasty by use of the timed up and go-test. BMC Musculoskelet Disord.

[9] Hofstede SN, Vliet Vlieland TP, van den Ende CH, Nelissen RG, Marang-van de Mheen PJ, van Bodegom-Vos L (2015). Variation in use of non-surgical treatments among osteoarthritis patients in orthopaedic practice in the Netherlands. BMJ Open.

[10] Füssenich W, Gerhardt DM, Pauly T, Lorenz F, Olieslagers M, Braun C, van Susante JL (2020). A comparative health care inventory for primary hip arthroplasty between Germany versus the Netherlands. Is there a downside effect to fast-track surgery with regard to patient satisfaction and functional outcome?. Hip Int.

[11] Heisel J, Jerosch J (2007). Rehabilitation nach Hüft- und Knieendoprothese [Rehabilitation After Hip and Knee Endoprosthesis]. Dt. Ärzte-Verlag, Cologne; 2007.

[12] Mooiweer Y, Roling L, Vugrin M, Ansmann L, Stevens M, Seeber GH (2024). Influence of patients' preoperative expectations on postoperative outcomes after total knee or hip arthroplasty: a systematic review. EFORT Open Rev.

[13] Brembo EA, Kapstad H, Van Dulmen S, Eide H (2017). Role of self-efficacy and social support in short-term recovery after total hip replacement: a prospective cohort study. Health Qual Life Outcomes.

[14] Stevens M, van den Akker-Scheek I, van Horn JR (2005). A Dutch translation of the Self-Efficacy for Rehabilitation Outcome Scale (SER): a first impression on reliability and validity. Patient Educ Couns.

[15] Hagger MS, Orbell S (2022). The common sense model of illness self-regulation: a conceptual review and proposed extended model. Health Psychol Rev.

[16] Nes LS, Segerstrom SC (2006). Dispositional optimism and coping: a meta-analytic review. Pers Soc Psychol Rev.

[17] Laferton JA, Oeltjen L, Neubauer K, Ebert DD, Munder T (2022). The effects of patients’ expectations on surgery outcome in total hip and knee arthroplasty: a prognostic factor meta-analysis. Health Psychol Revi.

[18] Bandura A (1977). Self-efficacy: toward a unifying theory of behavioral change. Psychol Rev.

[19] Lingard EA, Sledge CB, Learmonth ID (2006). Patient expectations regarding total knee arthroplasty: differences among the United States, United Kingdom, and Australia. J Bone Joint Surg Am.

[20] Kahlenberg CA, Nwachukwu BU, Schairer WW, Steinhaus ME, Cross MB (2017). Patient satisfaction reporting after total hip arthroplasty: a systematic review. Orthopedics.

[21] Mancuso CA, Sculco TP, Salvati EA (2003). Patients with poor preoperative functional status have high expectations of total hip arthroplasty. J Arthroplasty.

[22] Balck F, Kirschner S, Jeszenszky C, Lippmann M, Günther KP (2016). [Validity and reliability of the German version of the HSS expectation questionnaire on hip joint replacement]. Z Orthop Unfallchir.

[23] Waldrop D, Lightsey OR, Ethington CA, Woemmel CA, Coke AL (2001). Self-efficacy, optimism, health competence, and recovery from orthopedic surgery. J Couns Psychol.

[24] Bandura A (1997). Self-Efficacy: The Exercise Of Control. https://psycnet.apa.org/record/1997-08589-000.

[25] Mooiweer Y, Stevens M, van den Akker-Scheek I, Linngrön LL, Seeber GH (2025). The German Self-Efficacy for Rehabilitation Outcome Scale (SER-G) in patients undergoing primary total hip arthroplasty: a cross-cultural study into validity and reliability. J Public Health.

[26] Klässbo M, Larsson E, Mannevik E (2003). Hip disability and osteoarthritis outcome score - an extension of the Western Ontario and McMaster Universities Osteoarthritis Index. Scand J Rheumatol.

[27] de Groot IB, Reijman M, Terwee CB, Bierma-Zeinstra SM, Favejee M, Roos EM, Verhaar JA (2007). Validation of the Dutch version of the Hip Disability and Osteoarthritis Outcome Score. Osteoarthritis Cartilage.

[28] Blasimann A, Dauphinee SW, Staal JB (2014). Translation, cross-cultural adaptation, and psychometric properties of the German version of the hip disability and osteoarthritis outcome score. J Orthop Sports Phys Ther.

[29] Zhang J, Dashti SG, Carlin JB, Lee KJ, Moreno-Betancur M (2023). Should multiple imputation be stratified by exposure group when estimating causal effects via outcome regression in observational studies?. BMC Med Res Methodol.

[30] Mooiweer Y, Seeber GH, Brütt AL (2023). Influence of health system and patient characteristics on expectations and outcome in total hip arthroplasty patients in the Dutch-German border region: protocol for a mixed-methods prospective observational comparative study (hip across). BMJ Open.

[31] Buirs LD, Van Beers LW, Scholtes VA, Pastoors T, Sprague S, Poolman RW (2016). Predictors of physical functioning after total hip arthroplasty: a systematic review. BMJ Open.

[32] Okafor L, Chen AF (2019). Patient satisfaction and total hip arthroplasty: a review. Arthroplasty.

